# Machine Learning Model for Predicting Mortality Risk in Patients With Complex Chronic Conditions: Retrospective Analysis

**DOI:** 10.2196/52782

**Published:** 2023-12-28

**Authors:** Guillem Hernández Guillamet, Ariadna Ning Morancho Pallaruelo, Laura Miró Mezquita, Ramón Miralles, Miquel Àngel Mas, María José Ulldemolins Papaseit, Oriol Estrada Cuxart, Francesc López Seguí

**Affiliations:** 1 Research Group on Innovation, Health Economics and Digital Transformation Institut Germans Trias i Pujol Badalona Spain; 2 Hospital Germans Trias i Pujol Institut Català de la Salut Badalona Spain; 3 Direcció Clínica Territorial de Cronicitat Metropolitana Nord Institut Català de la Salut Badalona Spain; 4 Department of Geriatrics Hospital Germans Trias i Pujol Badalona Spain; 5 Direcció d’Atenció Primària Metropolitana Nord Institut Català de la Salut Badalona Spain; 6 Servei d’Atenció Primària Barcelonès Nord Institut Català de la Salut Barcelona Spain; 7 Chair in ICT and Health, Centre for Health and Social Care Research (CESS) University of Vic - Central University of Catalonia (UVic-UCC) Vic Spain

**Keywords:** machine learning, mortality prediction, chronicity, chromic, complex, artificial intelligence, complexity, health data, predict, prediction, predictive, mortality, death, classification, algorithm, algorithms, mortality risk, risk prediction

## Abstract

**Background:**

The health care system is undergoing a shift toward a more patient-centered approach for individuals with chronic and complex conditions, which presents a series of challenges, such as predicting hospital needs and optimizing resources. At the same time, the exponential increase in health data availability has made it possible to apply advanced statistics and artificial intelligence techniques to develop decision-support systems and improve resource planning, diagnosis, and patient screening. These methods are key to automating the analysis of large volumes of medical data and reducing professional workloads.

**Objective:**

This article aims to present a machine learning model and a case study in a cohort of patients with highly complex conditions. The object was to predict mortality within the following 4 years and early mortality over 6 months following diagnosis. The method used easily accessible variables and health care resource utilization information.

**Methods:**

A classification algorithm was selected among 6 models implemented and evaluated using a stratified cross-validation strategy with k=10 and a 70/30 train-test split. The evaluation metrics used included accuracy, recall, precision, *F*_1_-score, and area under the receiver operating characteristic (AUROC) curve.

**Results:**

The model predicted patient death with an 87% accuracy, recall of 87%, precision of 82%, *F*_1_-score of 84%, and area under the curve (AUC) of 0.88 using the best model, the Extreme Gradient Boosting (XGBoost) classifier. The results were worse when predicting premature deaths (following 6 months) with an 83% accuracy (recall=55%, precision=64% *F*_1_-score=57%, and AUC=0.88) using the Gradient Boosting (GRBoost) classifier.

**Conclusions:**

This study showcases encouraging outcomes in forecasting mortality among patients with intricate and persistent health conditions. The employed variables are conveniently accessible, and the incorporation of health care resource utilization information of the patient, which has not been employed by current state-of-the-art approaches, displays promising predictive power. The proposed prediction model is designed to efficiently identify cases that need customized care and proactively anticipate the demand for critical resources by health care providers.

## Introduction

Current health care systems are evolving toward a more patient-centered approach, ensuring continuity of care between primary and hospital care. Their goal is to work toward implementing strategies that favor integrated health and social care of excellent quality for individuals with chronic conditions, such as frailty, multimorbidity, or advanced illness, by prioritizing community care led by primary care teams to improve quality of life across the different stage of disease pathways—especially at the end of life [1-3]. This change in health care provision brings out a series of challenges such as predicting populations’ needs, selecting high-risk cases, and tracking illness trajectories, as well as tools to analyze which factors determine high patients’ needs in different stages, with the aim of optimizing resources, [4,5]. In Institut Català de la Salut (ICS), Catalonia’s main public health provider, this model has been designed and is being implemented by the Chronic Care Management Team in Barcelona’s North Metropolitan Area under the name “Community-Based Integrated Care Program for People With Complex Chronic Conditions” (ProPCC) [6] with promising results in terms of the decrease in emergency department attendance and hospitalizations [7]. In this scenario, the research team recognizes the necessity of developing supportive tools for care activities and data exploration. These tools aim to predict resource utilization for individual patients, their progression, and the variables influencing it. This initiative is geared toward enhancing resource planning within the program.

The exponential increase in the availability of health data is enabling the application of advanced statistics and artificial intelligence (AI) techniques for the health care system to evolve toward data-driven policy-making and develop decision support systems to improve efficiency in resource planning, prevent diagnoses evolutions, predict patient progression, and make screenings. However, most data currently being collected are not analyzed, either due to a lack of resources or professionals. AI and machine learning (ML) techniques are key to solving these challenges and automating the analysis of large volumes of medical data to reduce professionals’ workload and save resources [8,9]. Moreover, there is a severe shortage of professionals in the health care system, which these algorithms could help alleviate. They could greatly aid health care professionals by optimizing resource use and decreasing systemic gaps, with a particular focus on improving structure for the care of high-needs populations. The developed AI models must be robust, ensure privacy, and be easily generalized to other data and contexts in the health care field [10].

In the context of chronic care planning, predicting patient mortality in a short period of time could help the system better plan critical resources, most of which are spent in the last years of life, and provide closer accompaniment during the last phase of a patient's life [11]. In the medical field, predicting mortality has always been carried out in controlled contexts or in groups of patients with a specific disease. Most of this research has focused on predicting intensive care unit (ICU) mortality. Various studies have advocated the use of ML techniques over the use of logistic regression methods for prediction [12-14], achieving promising results, with an area under the receiver operating characteristic (AUROC) curve of 0.9 [13]. In a hemodialysis cohort, ML techniques were applied to predict death in patients who were critically ill (AUROC=0.86) [15].

In terms of predicting mortality focusing on certain diseases, there are multiple mortality risk scores for different diseases that have been used for a while in the Catalan Health system [16-18]. Regarding ML techniques, many of the models focused on diseases with a high prevalence in the population, such as cardiovascular diseases. ML algorithms such as the support vector machine-radial basis function (SVM-RBF) achieve good results in predicting the mortality onset of cardiovascular diseases [19-21]. Multiple studies use Cox proportional hazards regression models to predict the risk of death due to progressive chronic heart failure [22] and breast cancer [23]. In the case of patients with diabetes, some models have achieved good results in predicting the disease using SVM or NB algorithms, reaching an accuracy of 82% [11,24,25]. Research has prioritized identifying predictors of mortality related to diabetes, including hypercholesterolemia or serum albumin, which have been found to be significant predictors of death in patients undergoing hemodialysis [26,27].

With the COVID-19 pandemic, the need to generate prediction models at a population level has been brought to the forefront. The research paradigm has shifted from predicting mortality in a highly controlled population cohort with a specific clinical profile to the general population, with much more diverse clinical, socioeconomic, and demographic realities. The pandemic has provided the research field with a much higher volume of resources and data to develop models with acceptable accuracy. Recent studies have used deep learning (DL) models to predict the probability of entering the ICU or dying for patients diagnosed with COVID-19 (AUROC=0.84) [28]. Others have tried to predict the mortality rate of critically ill patients diagnosed with COVID-19, achieving promising results (AUROC=0.87) [29]. Other studies sought to predict mortality at the national level [30-32]. Regarding the prediction of mortality in patients with complex chronic conditions, AI and ML algorithms have not yet been used. Currently, only frailty indexes have been used to predict mortality risk in older populations [16].

In this context, this study aims to present an ML model applied to a cohort of patients with highly complex diagnoses, categorized as either a patient with complex chronic disease (CCP) or a patient with advanced chronic disease (ACP). These terms serve as indicators of health and social complexity as defined by the Catalan public health system [17]. The aim is to predict mortality, which is indicative of the severity of their condition by utilizing easily accessible retrospective data. The model aims to predict a future event, death, by incorporating different population variables to ascertain how their progression would be in each case.

## Methods

### Data Sources and Inclusion Criteria

The data set was obtained from the administrative database of Barcelonès Nord Primary Care Centers linked to the program and the Germans Trias i Pujol Hospital, a reference hospital for the region that covers a population of 1,448,812 people belonging to 70 municipalities. It includes the period between May 2018 and December 2021 (with a slowdown of inclusion due to the COVID-19 pandemic in 2020). The observation study was focused on individuals from the ProPCC cohort, including high-needs high-cost patients cataloged as CCP or ACP based on the Catalonia Department of Health’s risk stratification strategy [32,33]. This is a subanalysis from a retrospective observational study to estimate the impact of the introduction of ProPCC.

This study contains the main population characteristics of the patients, including age, sex, main diagnoses, complexity profiles (CCP or ACP), and adjusted mobility groups (ie, grupos de morbilidad ajustados, GMA). and place of habitual residence (home or nursing home). To enter the ProPCC program, patients had to meet one of the inclusion criteria for the program: (1) easily decompensated chronic conditions, (2) frequent visits to hospital emergency rooms for the same reason, (3) dementia with cognitive/behavioral handling challenges, (4) functional dependence with difficult handling, (5) difficulty accepting the loss of health, (6) polypharmacy with handling difficulty, and (7) advanced complex disease process or caregiver burden.

The database also contains information on the duration of time spent at home and the utilization of health care resources, encompassing primary care and hospital visits within the last 12 and 6 months, specifically, primary care visits and hospital services such as emergency department visits and acute hospitalizations.

### Ethical Considerations

This study was approved by the Medicines Research Ethics Committee of the Institut Universitari d’Investigació en Atenció Primària (IDIAP Jordi Gol; 22/084-P).

### Baseline Characteristics

This study included 264 patients identified as CCP (n=116, 43.9%) or ACP (n=148, 56.1%). The average age at inclusion in the study was 83.6 (SD 10.3) years, and 50% (n=132) of the patients were female. The mean time spent in the program since inclusion was 16.73 (SD 12.8) months. Most patients belonged to the highest adjusted morbidity group (ie, GMA; group 4, n=219, 83%), and 9.1% (n=24) lived in a nursing home. Most were included in the program because they had easily decompensated chronic conditions (n=145, 54.9%). The mean number of active health problems was 24.3 (SD 8.4). Table 1 summarizes all the variables used as predictors in the study. The variable death represents the variable to predict, while the rest act as predictors. Within the first 6 months after inclusion in the ProPCC program, 43.9% (n=116) of the patients in the study died. Subsequently, there were 171 (n=174, 65.9%) additional deaths in the remaining period.

**Table 1:** Clinical characteristics of the implementation cohort (N=264).

Main patient variables		Values
Age (years), mean (SD)		83.6 (10.3)
Female sex, n (%)		132 (50)
**Main clinical diagnoses, n (%)**		
	Respiratory	92 (34.8)
	Cardiological	112 (42.4)
	Cognitive disorder	86 (32.6)
	Diabetes mellitus 2	83 (31.4)
	Hypertension	158 (59.8)
	Musculoskeletal	156 (59.1)
	Renal failure	75 (28.4)
**Complexity profiles, n (%)**		
	CCP^a^	117 (44.3)
	ACP^b^	147 (55.7)
**Adjusted morbidity groups, n (%)**		
	Group 4	156 (83)
	Group 3	30 (15.9)
	Group 1-2	2 (1.3)
**Program inclusion criteria, n (%)**		
	Chronic conditions easily decompensated	145 (54.9)
	Frequent visits to the emergency room for the same reason	32 (12.1)
	Dementia with cognitive/behavioral management difficulties	23 (8.7)
	Hard-to-handle functional dependency	53 (20.1)
	Difficulty accepting the loss of health	31 (11.7)
	Polypharmacy with complex handling	48 (18.2)
	Advanced complex disease process	51 (19.3)
	Caregiver burden	31 (11.7)
**Consumption of previous care resources, mean (SD)**		
	Family doctor visit 12 months prior	12.4 (17)
	Family doctor visit 6 months prior	8.2 (8.3)
	Nursing visit 12 months prior	13.1 (20.7)
	Nursing visit 6 months prior	9.3 (11.9)
	Social worker visit 12 months prior	1.5 (3.7)
	Social worker visit 6 months prior	1 (2.7)
	Continued care visit 12 months prior	1.2 (6.6)
	Continued care visit 6 months prior	0.7 (3.7)
**Consumption of previous hospital care resources, mean (SD)**		
	Home hospitalization 12 months prior	0.2 (0.6)
	Home hospitalization 6 months prior	0.1 (0.4)
	Acute hospitalization 12 months prior	1.1 (1.5)
	Acute hospitalization 6 months prior	0.7 (1.1)
	Visit to the emergency room 12 months prior	2.3 (2.6)
	Visit to the emergency room 6 months prior	1.4 (1.7)
**Death (outcome variable), n (%)**		
	Within the first 6 months of program entry	114 (43.4)
	Throughout the rest of the study period	171 (64.7)

^a^CCP: patient with complex chronic disease.

^b^ACP: patient with advanced chronic disease.

### Mortality in High-Risk CCP and ACP

**Figure 1:**
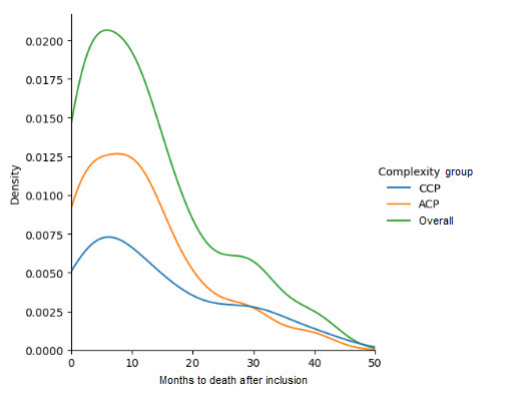
Death distribution over the study period (overall and by complexity groups). ACP: patient with advanced chronic disease; CCP: patient with complex chronic disease.

Patients entering the ProPCC program had highly complex and chronic clinical profiles. This caused the death rate to be much higher than in any other health program, with a high probability of having died in the 2 years following the start of the program. Among the 264 patients who were monitored in the ProPCC program during the 48 months of study, 171 (64.7%) died. The case fatality ratio over the first 6 months in the program was 43.2% (n=114). The average patient died after 12.61 (SD 10.8) months. The death distribution over the study time period representation is shown in Figure 1. Table 2 contains a description of the mortality variable, overall and by complexity subgroups. The study only considers the first 4 years to predict patient mortality. The objective of this analysis was to predict which patients would die within the first 48 months and who would experience premature death within the first 6 months. By definition, a patient with complexity transitions from CCP to ACP. Therefore, it was highly probable that an ACP would die within the first year since the complex diagnosis but less likely to be the case for a CCP.

**Table 2:** Description of ProPCC^a^ program prediction variables (mortality up to 4 years).

Prediction variables	Value, n (%)	Mean	SD	Min	Q1	Median	Q3	Max
Months until death overall	171 (64.8)	12.6	10.8	0	4	10	18	43
Months until death for CCP^a^	68 (58% CCP, 39% deaths, 25.8% global)	14.2	11.9	0	4.8	10	22.5	43
Months until death for ACP^b^	103 (70% ACP, 60% deaths, 39% global)	11.6	10	0	3.5	10	16.5	40

^a^CCP: patient with complex chronic disease.

^b^ACP: patient with advanced chronic disease.

### Data Processing

There were no missing values in the database. The numerical variables (ie, age, resource consumption, program inclusion criteria) were normalized and scaled using a min-max scaler. The rest of the categorical variables underwent a one-hot encoding transformation. The classification variable was binarized from the “months until death” variable based on the different experimentation use cases defined in the “Experimental Setup” section. Only retrospective information (up to 1 year prior to inclusion in the ProPCC program) was used to predict death in the following months (48 months or 4 years). No data collected during the response time were used for prediction.

### Model Construction and Evaluation

The developed method aimed to find classification algorithms capable of predicting the mortality of patients with high complexity using retrospective variables available within the health provider's information systems. This approach aimed to use these variables as predictors without the need to conduct surveys or use external data repositories. The ML algorithms we used included SVM with a linear kernel (SVM-Lin) with a one-versus-one decision function, SVM with RBF as a kernel (SVM-RBF, regularization parameter c=20), a k-nearest-neighbors classifier (KNN, k=25), decision tree classification (Tree), an Extreme Gradient Boosting (XGBoost) classifier, and a Gradient Boosting (GRBoost) classifier (learning_rate=0.25). All parameters were optimized to identify the best parameterization strategy. All models were implemented using the open-source Python ML library *Scikit-learn* and *dmlc-XGBoost*. A stratified cross-validation (CV) strategy with k=10 and a 70/30 train-test split was used to evaluate and validate the models. Evaluation metrics are averaged over all k-fold CVs.

The metrics used to evaluate the models were accuracy, which measured how many observations, both positive and negative, were correctly classified; recall and precision, extracting the ratio of death patients correctly classified as dead; *F*_1_-score, which measures the harmonic mean between precision and recall; and AUROC curve, which provides an aggregate measure of performance across all possible classification thresholds.

To obtain the algorithm that yielded the best results in the different experiments, predicting such a critical label as death, we focused on maximizing the recall, and subsequently, the *F*_1_-score (reducing the number of false negatives). The aim of the model was to save resources by identifying patients with a higher risk of death, and hence more severe health conditions, to participate in the ProPCC study. Thus, it was crucial to reduce the number of false negatives, as this could imply less care for the patient than necessary.

However, emphasizing precision was not as vital since it could result in offering more services to individuals with lower mortality risk. Participation in the program did not harm the patient; thus, it was not a critical prediction for health. Nonetheless, the primary objective was to minimize the number of patients who are flagged as likely to die but do not; hence, our emphasis was more on *F*_1_-score than overall accuracy. Employing this methodology and deriving the mean with a 10-fold CV yielded more robust results, balancing the classes and generating results less impacted by the minor imbalance in the data set. The Cochran Q test was performed to assess the robustness of the results.

Once we identified the algorithm with the best results in terms of metrics, we used the open-source Python library *Streamlit* to develop a user-friendly web app with a good user interface and user experience for health care professionals.

### Experimental Setup

This study was divided into 3 experiments based on patients’ period of death. All the experiments used the same models, parametrizations, and variables described in Table 1 (ie, age and sex, clinical diagnostics, complexity profile, morbidity profile, program inclusion criteria, and consumption of primary care and hospital care resources in the 6 and 12 months before inclusion). The objective was to detect which model more accurately predicted mortality in each experiment, using the same accessible variables. These experiments were structured as binary classifications (1=death, 0=survival). The first experiment aimed to evaluate the algorithms' ability to predict patient mortality throughout the study period (48 months, 4 years). In this scenario, the binary outcomes were as follows: 1=patient death (n=171, 64.8%) and 0=patient survival (n=93, 35.2%). The second scenario was designed to evaluate the algorithms' predictive ability to detect premature mortality (within 6 months). The binary results were as follows: 1=patient death within the first 6 months of the study (n=58, 22%) and 0=patient survival after 6 months (n=206, 78%). The third scenario aims to evaluate the algorithm’s ability to classify which patients will likely die prematurely (within 6 months) and which will likely die later to check for differences. The binary results were as follows: 1=patient death within the first 6 months of the study (n=58, 34%) and 0=patient death after 6 months (n=113, 66%).

## Results

### Overview

**Figure 2:**
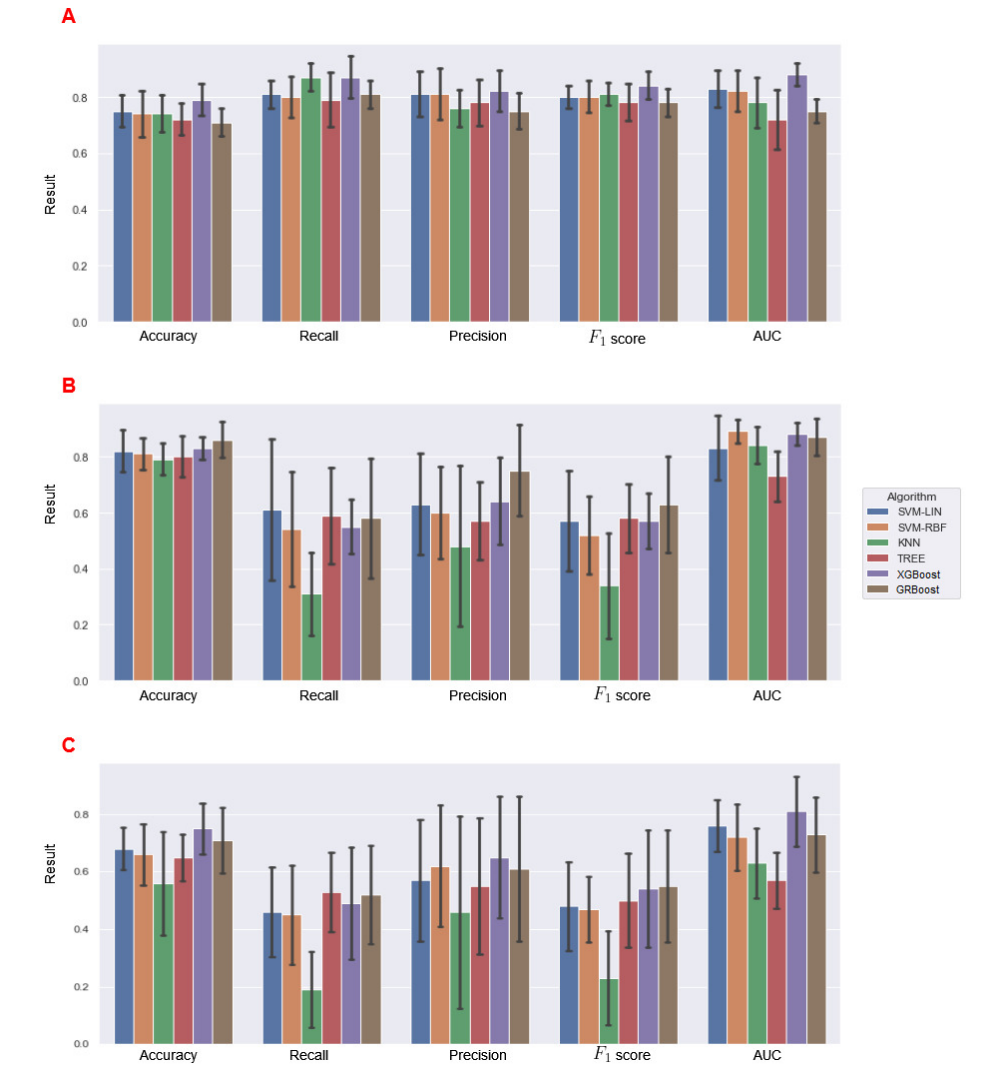
Performance metrics comparison of the different models in the different use case scenarios. (A: mortality in the entire study period, 48 months; B: premature death, 6 months; C: premature death versus dead). AUC: area under curve; GRBoost: Gradient Boosting; KNN: k-nearest neighbor; Lin: linear kernel; RBF: radial basis function; SVM: support vector machine; Tree: decision tree; XGBoost: Extreme Gradient Boosting.

The averaged results of all models for the stratified k-fold CV, including all metrics for each algorithm in the different use case scenarios, are presented in Table 3 and Figure 2 to visualize the comparison between the different models.

**Table 3:** Performance results of the algorithms in the different use case scenarios.

Model		Accuracy	Recall	Precision	*F*_1_-score	AUC^a^
**Mortality prediction (48 months), mean (SD)**						
	SVM^b^-Lin^c^	75 (7)	81 (6)	81 (10)	80 (5)	83 (8)
	SVM-RBF^d^	74 (10)	80 (9)	81 (11)	80 (7)	82 (9)
	KNN^e^	74 (8)	87 (6)	76 (8)	81 (5)	78 (11)
	Tree^f^	72 (7)	79 (12)	78 (10)	78 (8)	72 (13)
	XGBoost^g^	79 (7)	87 (7)	82 (9)	84 (6)	88 (5)
	GRBoost^h^	71 (6)	81 (6)	75 (8)	78 (6)	75 (5)
**Early mortality prediction (within 6 months), mean (SD)**						
	SVM-Lin	82 (9)	61 (31)	63 (22)	57 (22)	83 (14)
	SVM-RBF	81 (7)	54 (25)	60 (20)	52 (17)	89 (5)
	KNN	79 (7)	31 (18)	48 (35)	34 (23)	84 (8)
	Tree	80 (9)	59 (21)	57 (17)	58 (15)	73 (11)
	XGBoost	83 (5)	55 (12)	64 (19)	57 (12)	88 (5)
	GRBoost	86 (8)	58 (26)	75 (20)	63 (21)	87 (8)
**Early mortality prediction vs late mortality (within and after 6 months), mean (SD)**						
	SVM-Lin	68 (9)	46 (19)	57 (26)	48 (19)	76 (11)
	SVM-RBF	66 (13)	45 (21)	62 (26)	47 (14)	72 (14)
	KNN	56 (22)	19 (16)	46 (41)	23 (20)	63 (15)
	Tree	65 (10)	53 (17)	55 (29)	50 (20)	57 (12)
	XGBoost	75 (11)	49 (24)	65 (26)	54 (25)	81 (15)
	GRBoost	71 (14)	52 (21)	61 (31)	55 (24)	73 (16)

^a^AUC: area under the curve.

^b^SVM: support vector machine.

^c^Lin: linear kernel.

^d^RBF: radial basis function.

^e^KNN: k-nearest neighbor.

^f^Tree: decision tree.

^g^XGBoost: Extreme Gradient Boosting.

^h^GRBoost: Gradient Boosting.

### Model Performance

The performance of the models varied significantly depending on the use case. The best results were obtained when identifying patient mortality during the entire study period.

**Figure 3:**
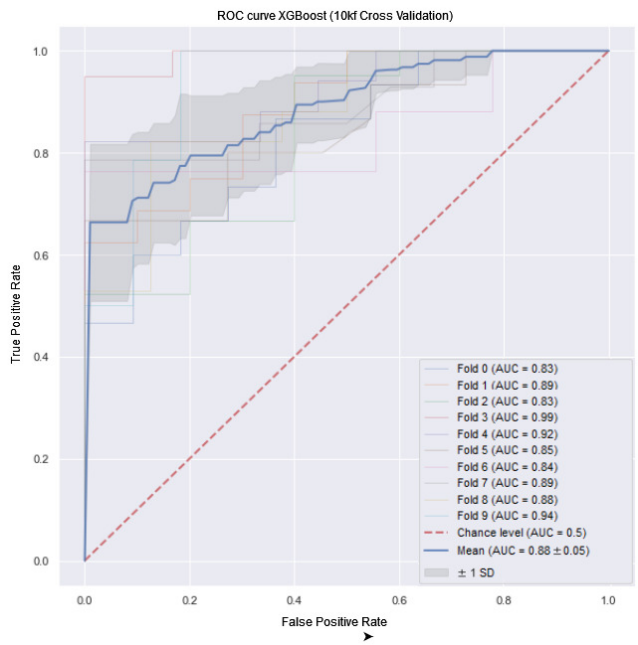
Area under the receiver operating characteristic (AUROC) curve for the algorithm with the best performance. Extreme Gradient Boosting (XGBoost) averaged over the k-fold cross-validation (CV, k=10). First experiment (mortality prediction over the entire period: 10 k-fold area under the curve (AUC)=0.88, SD 0.05).

In the first experiment, the XBoost algorithm obtained the best results in all metrics. The results were obtained by averaging partial results from a 10 k-fold CV. The SD was small for all metrics, demonstrating robustness across different partial results. XGBoost achieved the best result, especially in the most critical metrics (recall=87%, *F*_1_-score=84%, and AUROC=88%). The average Cochran Q test results obtained in different folds were significant in training (Q=137.7, *P*<.001) but not significant in testing (Q=5.3, *P=*.43). Regarding testing, there was no significant difference in the accuracy of the different models. However, in terms of the other metrics, the XGBoost model was best suited for predicting patient mortality throughout the study period (48 months, 4 years). Figure 3 shows the AUROC curve of this experiment,

In the second and third experiments, the results are worse. In both cases, the SDs obtained were higher than in the first experiment, indicating that the models were not as robust and were highly sensitive to the cases found in each fold. GRBoost with a 25% learning rate obtained good results in predicting premature deaths (6 months after data collection), especially in terms of accuracy (86%) and AUC (87%), with low SDs. However, in terms of recall (58%), precision (75%), and *F*_1_-score (63%), the results were less promising and highly sensitive to the fold used (high SDs). SVM-RBF and XGBoost also obtained good results in AUC and accuracy. The average results of the Cochrane Q test obtained in the different folds were significant in the training set (Q=113.9, *P*<.001) but not significant in the testing set (Q=7.1, *P=*.36). Although the results were not as robust and promising as in the first use case, the overall accuracy and AUC were very high in different models, demonstrating a correlation between the predictors and premature death.

In use case scenario 3, where we aimed to create a model capable of distinguishing between participants who would die prematurely and those who would die during the rest of the study period (without considering survivors), we obtained the poorest results. XGBoost achieved the highest accuracy (75%) and AUC (81%). However, in the other metrics, there was considerable variability across all models, demonstrating that the results were highly sensitive to the fold used. The top *F*_1_-score (55%) was obtained by GRBoost, although it was much lower than the results obtained in the previous use cases.

As depicted in Figure 2, XGBoost usually obtained the best results in all 3 scenarios**.** GRBoost and SVM-RBF also performed well in different scenarios.

## Discussion

### Principal Findings

Our research shows that ML and AI models can predict mortality in CCP and ACP with a high degree of accuracy (up to 89%) using easy-to-obtain real-life predictor variables not typically used in this type of experiment. This study illustrates that incorporating these variables has the potential to enhance analysis and accurately predict mortality among patients with significant health and social chronic needs. Previous studies showed similar results [12,14,26,27,29] or better ones [17,22,28,30] in terms of metrics. However, no literature has been found that specifically refers to predicting mortality within the high-need high-cost patient population. Most models for predicting mortality among chronic patients focused on cohorts with a specific diagnosis or establishing basic associations among variables linked to mortality. We have not found a similar use case in the literature that seeks to predict mortality in an identifiable yet heterogeneous group. Additionally, no identified model has been capable of predicting mortality using the consumption of primary care and hospital care resources.

The comparison was carried out with cases of prediction for specific diseases [17,22] or controlled contexts such as ICUs [12,14] or the COVID-19 pandemic outbreak [26-30].

Observing the outcomes, studies predicting mortality in ICUs obtain metrics akin to this study’s findings, whereas those addressing specific pathologies present higher metrics. Unlike other death prediction models, the proposed model not only uses patient clinical data such as diagnosis or morbidity but also proposes a framework utilizing medical care resource consumption data during the previous year (visits to different clinical specialties) and sociodemographic health-related variables. These variables include difficulty accepting health loss, dementia, caregiver burden, functional dependence, polypharmacy, or frequent consultations for the same reason. Incorporating these variables as predictors alongside previously established predictive variables resulted in robust outcomes, accurately predicting cases where patients may face mortality. This model can be used to screen patients who will enter the ProPCC program when scaled to all health care areas, aiding in optimizing critical health care resources and supporting patients at a higher risk of mortality.

### Limitations

This study is subject to several limitations that warrant careful consideration. First, it is important to acknowledge the relatively small size of the cohort. The limited number of patients included in our analysis suggests that there is ample room for improvement to bolster the robustness of the findings, particularly through validation in a larger and more diverse cohort. It is worth noting that the incorporation of a 10-fold CV methodology was employed to enhance the reliability of the results and demonstrate the model’s predictive capability. However, the inclusion of a larger sample size would provide more compelling evidence and strengthen the generalizability of our study's outcomes.

Second, an inherent limitation of the study lies in the potential unbalance present within the data sets. This may introduce errors, particularly when predicting mortality, especially in scenarios 2 and 3 where the occurrence of mortality within the sample was relatively lower. Consequently, the model may not adequately capture the intricate patterns and factors associated with mortality in these specific scenarios. It is advisable to address this issue by employing appropriate techniques such as data set balancing if the data set is large enough, thereby potentially improving the model's performance and accuracy in predicting outcomes.

Third, it is essential to consider the quality of data derived from electronic health systems, particularly within noncontrolled environments. While the data set utilized herein exhibited a high standard of coding quality, which was meticulously ensured by a team of proficient professionals, it is crucial to recognize applying these models to uncured data sources within the health care system may yield less favorable results. The inherent variability and potential inconsistencies in data coding across diverse sources may substantially impact the model's performance and its ability to generalize findings to real-world scenarios.

In summary, this study has acknowledged several notable limitations, including the modest cohort size, potential errors arising from data set unbalance, and potential challenges associated with applying the models to real-world data sources characterized by lower data quality. These limitations should be duly considered when interpreting our study findings, and further endeavors should be undertaken to enhance the robustness and applicability of the models.

### Conclusion and Future Work

This study was developed under the hypothesis that mortality among high-need high-cost patients could be predicted in advance using easily accessible real-life patient variables, including clinical, economic resource use, and demographic variables. With this premise, we used an agnostic approach to develop and test a series of ML algorithms to evaluate predictive capacity in a highly controlled cohort of CCP and ACP. The models achieved robust performance in terms of AUC (88%), accuracy (87%), and *F*_1_-score (84%), indicators that are particularly important in this prediction context.

Our model not only offers a high degree of prediction accuracy but also provides graphs with the importance of each variable in predicting the risk of mortality. The algorithm is encapsulated in a web app developed with the open-source Python library *Streamlit* to improve usability, transportability, and interpretability for medical professionals. This facilitates more efficient utilization of technology resources and enables the system to predict clinical resource usage, thereby offering better support to patients in the end-of-life stage. This type of tool enables process automation and analysis of large volumes of data, while also allowing the seamless transfer of the same infrastructure to different contexts or units.

Regarding future work, there are relevant challenges such as increasing the number of patients and including new clinical and analytical variables that can act as predictors. Our study cohort exhibits high-quality data, meticulously reviewed by multiple professionals to ensure that there are no errors or missing values. In comparison, primary databases, despite containing a significantly larger patient pool, tend to have lower-quality data. Future objectives include testing the model using this data set and a much larger number of patients, not just from the high-need profile. Deep-learning models could also further refine the results.

## Abbreviations

ACP: patient with advanced chronic disease
AI: artificial intelligence
AUC: area under the curve
AUROC: area under the receiver operating characteristic
CCP: patient with complex chronic disease
CV: cross-validation
DL: deep learning
GMA: grupos de morbilidad ajustados
GRBoost: Gradient Boosting
ICS: Institut Català de la Salut
ICU: intensive care unit
IDIAP: Institut Universitari d’Investigació en Atenció Primària
KNN: k-nearest-neighbor
ML: machine learning
ProPCC: Community-Based Integrated Care Program for People With Complex Chronic Conditions
RBF: radial basis function
SVM: support vector machine
XGBoost: Extreme Gradient Boosting

## Data Availability

The data supporting this study’s findings belong to the Gerència Territorial Metropolitana Nord of the Catalan Health Institute (ICS). Restrictions apply to the availability of these data, which were used after signing a data processing agreement and an ethical agreement that complies with the requirements of the current legal framework.
