# Correction: Machine Learning Model for Predicting Mortality Risk in Patients With Complex Chronic Conditions: Retrospective Analysis

**DOI:** 10.2196/58453

**Published:** 2024-03-21

**Authors:** Guillem Hernández Guillamet, Ariadna Ning Morancho Pallaruelo, Laura Miró Mezquita, Ramón Miralles, Miquel Àngel Mas, María José Ulldemolins Papaseit, Oriol Estrada Cuxart, Francesc López Seguí

**Affiliations:** 1 Research Group on Innovation, Health Economics and Digital Transformation Institut Germans Trias i Pujol Badalona Spain; 2 Hospital Germans Trias i Pujol Institut Català de la Salut Badalona Spain; 3 Direcció Clínica Territorial de Cronicitat Metropolitana Nord Institut Català de la Salut Badalona Spain; 4 Department of Geriatrics Hospital Germans Trias i Pujol Badalona Spain; 5 Direcció d’Atenció Primària Metropolitana Nord Institut Català de la Salut Badalona Spain; 6 Servei d’Atenció Primària Barcelonès Nord Institut Català de la Salut Barcelona Spain; 7 Chair in ICT and Health, Centre for Health and Social Care Research (CESS) University of Vic - Central University of Catalonia (UVic-UCC) Vic Spain

In “Machine Learning Model for Predicting Mortality Risk in Patients With Complex Chronic Conditions: Retrospective Analysis” (Online J Public Health Inform 2023;15:e52782) the authors noted one error.

The Corresponding Author’s address previously appeared as:

Chair in Palliative Care, Centre for Health and Social Care Research

University of Vic - Central University of Catalonia

Carrer Miquel Martí i Pol, 1

Vic, 08500

Spain

This has been changed to:

Chair in ICT and Health, Centre for Health and Social Care Research (CESS)

University of Vic - Central University of Catalonia (UVic-UCC)

Carrer Miquel Martí i Pol, 1

Vic, 08500

Spain

The correction will appear in the online version of the paper on the JMIR Publications website on March 21, 2024, together with the publication of this correction notice. Because this was made after submission to PubMed, PubMed Central, and other full-text repositories, the corrected article has also been resubmitted to those repositories.

